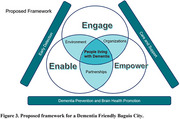# Towards Dementia‐Friendly Communities; The Case of Baguio City

**DOI:** 10.1002/alz70860_100026

**Published:** 2025-12-23

**Authors:** Florilyn Joyce Chulsi Bentrez

**Affiliations:** ^1^ Baguio General Hospital and Medical Center, Baguio City, Benguet, Philippines

## Abstract

**Background:**

The increasing prevalence of dementia among the elderly population poses a significant public health challenge, particularly in urban areas like Baguio City, Philippines. With a growing number of individuals living with dementia, there is an urgent need to foster dementia‐ friendly communities (DFCs) that can enhance the quality of life for those affected. This study aims to assess the current status of dementia‐friendliness in Baguio City, the barriers and facilitators, and community recommendations. Ultimately, the main goal is to create an action framework dedicated to promote a dementia‐friendly Baguio City.

**Method:**

A qualitative approach was employed, utilizing in‐depth interviews with 16 stakeholders, including healthcare providers, family members, caregivers, and community leaders. Participants were selected through purposive sampling to ensure diverse perspectives, and data were analyzed thematically to identify key concepts.

**Result:**

The study revealed that Baguio City currently lacks a dedicated dementia ordinance and formal support programs specifically for individuals living with dementia. Awareness level among the public was low, with prevalent misconceptions equating dementia with normal aging. While a newly established daycare program for persons living with dementia was noted as a positive initiative, challenges such as late detection, out‐of‐pocket healthcare costs, and inadequate infrastructure for individuals with dementia were prevalent. The community's attitude towards people living with dementia is generally supportive. With regards to nursing homes, some families recognize the need, but some express mistrust toward them. Recommendations include developing dedicated dementia policies, enhancing community awareness strategies, and improving local healthcare and support services.

**Conclusion:**

By implementing the action framework derived from this study, stakeholders can collaboratively work towards establishing a dementia‐friendly community that prioritizes early detection, comprehensive care, and support while also promoting dementia prevention initiatives. Creating such an environment is critical for improving the well‐being and quality of life for the growing elderly population in Baguio City, ultimately ensuring that individuals living with dementia are recognized, respected, and supported within their communities. Through collective effort and commitment, Baguio City can serve as a model for other areas striving to foster inclusivity and compassion for those affected by dementia.